# The Roles of *Epinephelus coioides* miR-122 in SGIV Infection and Replication

**DOI:** 10.1007/s10126-021-10023-w

**Published:** 2021-02-11

**Authors:** Hong-Yan Sun, Yu-Ling Su, Pin-Hong Li, Jia-Yang He, He-Jia Chen, Gang Wang, Shao-Wen Wang, Xiao-Hong Huang, You-Hua Huang, Qi-Wei Qin

**Affiliations:** Joint Laboratory of Guangdong Province and Hong Kong Regions on Marine Bioresource Conservation and Exploitation, Guangdong Laboratory for Lingnan Modern Agriculture, College of Marine Sciences, South China Agricultural University, Guangdong Province 510642 Guangzhou, People’s Republic of China

**Keywords:** *Epinephelus coioides*, miR-122, SGIV, Apoptosis

## Abstract

In mammals, mature miR-122 is 22 nucleotides long and can be involved in regulating a variety of physiological and biological pathways. In this study, the expression profile and effects of grouper *Epinephelus coioides* miR-122 response to Singapore grouper iridovirus (SGIV) infection were investigated. The sequences of mature microRNAs (miRNAs) from different organisms are highly conserved, and miR-122 from *E. coioides* exhibits high similarity to that from mammals and other fish. The expression of miR-122 was up-regulated during SGIV infection. Up-regulation of miR-122 could significantly enhance the cytopathic effects (CPE) induced by SGIV, the transcription levels of viral genes (MCP, VP19, LITAF and ICP18), and viral replication; reduce the expression of inflammatory factors (TNF-a, IL-6, and IL-8), and the activity of AP-1 and NF-κB, and miR-122 can bind the target gene p38α MAPK to regulate the SGIV-induced cell apoptosis and the protease activity of caspase-3. The results indicated that SGIV infection can up-regulate the expression of *E. coioides* miR-122, and up-regulation of miR-122 can affect the activation of inflammatory factors, the activity of AP-1 and NF-κB, and cell apoptosis to regulate viral replication and proliferation.

## Introduction

MicroRNAs (miRNAs), approximately 18–25 bp nucleotides, are small non-coding RNAs that participate in gene transcription and expression by regulating the target genes (Pillai et al. 2007; Sticht et al. 2018; Yurikova et al. 2019; Jangra et al. 2010). They were first discovered in *Caenorhabditis elegans* in 1990 and have quickly been recognized as fundamental components of systems that regulate gene expression (Lee et al. 1993). Today, miRNAs have been shown to be widespread in metazoans, plants, invertebrates, and vertebrates and can modulate many fundamental biological processes, such as cell growth, proliferation, differentiation, immuno-inflammatory responses, and apoptosis (Bushati and Cohen 2007; Bartel 2004).

MiR-122, a 22-nucleotide miRNA, was first discovered in the liver of adult mice (Lagos-Ouintana et al. 2002). Subsequently, the roles of miR-122 were elucidated. In mammals, miR-122 is one of the most abundant miRNAs in the liver, and it is also expressed in other tissues (Li et al. 2015; Laudadio et al. 2012; Xu et al. 2010; Bandiera et al. 2015; Fong et al. 2015). MiR-122 can regulate different pathways to participate in the following functions: regulating p53/Akt signaling, miR-122 can be involved in chemotherapy-induced apoptosis in cutaneous T cell lymphoma (Manfe et al. 2012); regulating the Hsp-70-dependent NF-κB pathway by targeting FOXO3, it can inhibit ischemic neuronal death (Guo et al. 2018); inhibiting the TLR4/MyD88/NF-κB p65 signaling pathway, it can reduce lipid accumulation and inflammation in L02 cells induced by OA (Hu et al. 2019); targeting IGF1R and regulating the PI3K/Akt/mTOR/p70S6K pathway, it can inhibit tumors such as breast cancer cell proliferation and tumorigenesis (Wang et al. 2012). MiR-122 can be involved in regulating the viral infection: it is essential for the production of hepatitis C virus (HCV) infection (Jangra et al. 2010); it can reduce the replication of Hepatitis B virus (HBV), indicating that miR-122 may represent a potential therapy for treatment of HBV infection (Song et al. 2015); it can repress the translation and replication of Borna disease virus (BDV) and the espression of IFNα and IFNβ to regulate the interaction between virus and host (Qian et al. 2010).

In recent years, the roles of miR-122 in lower vertebrates, such as fish, have been subsequently explored. In *Danio rerio*, anti-miR-122 can suppress zebrafish liver development (Laudadio et al. 2012); in *Oreochromis niloticus*, miR-122 can promote genetically modified farmed tilapia by directly targeting metallothionein genes to prevent the oxidative defense of the liver against cadmium (Qiang et al. 2017), and in *Oncorhynchus mykiss*, miRNA-122b promotes the expression of lipogenic genes and inhibits the expression of lipolytic genes (Mennigen et al. 2012, 2014). However, few reports have focused on the role miR-122 in fish during viral infection.

Grouper *Epinephelus coioides* is an economically important maricultured fish in China and Southeast Asian counties. Singapore grouper iridovirus (SGIV), a large cytoplasmic DNA virus, can cause high mortality rates in groupers and substantial economic losses to the aquaculture industry (Song et al. 2004). Only one type of miR-122 was found in *E. coioides*, and the miR-122 sequence of *E. coioides* was as that of miR-122a. In this study, the expression profile of *E. coioides* miR-122 during SGIV infection, and its roles in AP-1 and NF-κB activation, and SGIV-induced cell apoptosis were investigated.

## Materials and Methods

### Cell and Virus

Grouper spleen (GS) cells and fathead minnow (FHM) cells were propagated in our laboratory with Leibovitz’s L-15 medium containing 10% fetal bovine serum (Gibco, USA) at 28 ℃. The SGIV used in this study was originally separated and propagated by our laboratory as previously described (Qin et al. 2002). Virus-infected cells were collected and stored at − 80 ℃ for further analysis. Virus was titrated by the 50% tissue culture infectious dose (TCID_50_) assay, and an m.o.i. of 0.1 was used for the experiments (Ni et al. 2017).

### MiR-122 and Plasmid Transfection

MiR-122 mimics (miR-122) and control mimics (miR-con) were from RiboBio (RiboBio, China). MiR-122 or miR-con (100 nM) was transfected into FHM/GS cells by Lipofectamine RNAiMAX (Invitrogen, USA) according to the manufacturer’s instructions. The plasmid was transfected by Lipofectamine 2000 (Invitrogen) as previously described (Ni et al. 2017).

### RNA Extraction and cDNA Synthesis

Total RNA was extracted by TRIzol reagent (Invitrogen, USA). ReverTra Ace kit (TOYOBO, Japan) was used to prepare cDNA according to the manufacturer’s instructions. MiR-122 bulge-loop reverse transcription was performed with a specific customized miRNA RT kit (RiboBio, China). RNA (1.5 μg) was denatured at 70 ℃ for 10 min. Twenty microliter reaction contains 4 μl of 5 × RT buffer, 5 pmol miR-122 bulge-loop RT primers, 1 μl of RT polymerase, and 1.5 μg RNA. The reaction was performed inreverse transcription system: 42 ℃, 60 min; 72 ℃, 10 min.

### Real-Time Quantitative PCR

miR-122 level was determined by real-time quantitative PCR (qPCR) using a bulge-loop qPCR starter kit (RiboBio, China) and performed on Q5 detection system (Thermo, USA). A 10 μl reaction contained 5 μl SYBR qPCR mix, 0.4 μl forward and reverse bulge-loop miRNA primers (miR-122, U6, and β-actin primers were provided by RiboBio, China), 3.2 μl ddH_2_O, and 1 μl diluted miR-122 specific cDNA. The PCR program was 95 °C, 1 min; (95 °C, 15 s; 60 °C, 30 s) for 40 cycles. Small nuclear RNA U6 and β-actin were used as a reference in this study (Liu et al. 2012). Host genes and viral genes were determined by qPCR. A 10 μl reaction containing 5 μl SYBR qPCR mix, 0.4 μl forward and reverse primers (Table 1), 3.2 μl ddH_2_O, and 1 μl diluted cDNA was performed in the PCR program: 95 °C, 1 min (95 °C, 15 s; 60 °C, 15 s; 72 °C, 45 s) for 45 cycles. β-Actin and 18S rDNA were used as a reference, respectively (Guo et al. 2016; Huang et al. 2016a, b; Su et al. 2019). PCR amplification was performed in quadruplicate wells. The expression of the genes was calculated with the 2^−△△Ct^ method (Su et al. 2019).

**Table 1:** Primers used in this study

Primer	Sequence (5′ to 3′)
TNFα-F	GTGTCCTGCTGTTTGCTTGGTA
TNFα-R	CAGTGTCCGACTTGATTAGTGCTT
IL-6-F	CTCTACACTCAACGCGTACATGC
IL-6-R	TCATCTTCAAACTGCTTTTCGTG
IL-8-F	GCCGTCAGTGAAGGGAGTCTAG
IL-8-R	ATCGCAGTGGGAGTTTGCA
Bax-F	TGTGCGACCCAAATACCAAGAGG
Bax-R	AAGTAGAACAGTGCAACCACCCTGC
p53-F	GGAGGAAAACAGCACCAAGACGC
p53-R	CCACGAACATGCAGAACAAACACG
β-actin-F	TGCTGTCCCTGTATGCCTCT
β-actin-R	CCTTGATGTCACGCACGAT
18S-qRT-F	ATTGACGGAAGGGCACCACCAG
18S-qRT-R	TCGCTCCACCAACTAAGAACGG
β-actin-F	TGCTGTCCCTGTATGCCTCT
β-actin-R	CCTTGATGTCACGCACGAT
p38α-UTR-F1	GACTAGTTGAGTTTTGAGCCACCGTCGTT
p38α-UTR-R1	CAAGCTTCGGCAGGCACTTGAATGTC
p38α-mUTR3-F1	CCAGGAATGTGAGGTTAAAGACTATCAGTCTCTTGT
p38α-mUTR3-R1	CTTTAACCTCACATTCCTGGAAAACAAAAAGAT

### The Prediction of Target Genes

3′ untranslated region (UTR) regions of *E. coioides* immune-related genes from NCBI database and the *E. coioides* ESTs sequence database in our laboratory were analyzed. RNAhybrid (https://bibiserv.cebitec.uni-bielefeld.de/rnahybrid) was used to predict the potential targets of miR-122 and energy threshold was set at less than or equal to − 15 kcal/mol.

### Construction of Target-Contained Luciferase Plasmids

Wild-type UTR of p38α MAPK containing the putative target of miR-122 was amplified with primers p38α-UTR-F1/p38α-UTR-R1 (Table 1). Mutant UTR (mUTR) with missense mutation of predicted target sites was amplified using the primer p38α-mUTR3-F1/p38α-mUTR3-R1. The two UTR fragments were cloned into the pMIR-REPORT luciferase vector (Ambion, USA), respectively, between the HindIII and Spel restriction enzyme sites. Recombinant plasmids were verified by DNA sequencing (Invitrogen, Guangzhou).

### Hoechst 33342 DNA Staining

FHM cells were stained using fluorescent DNA Hoechst 33342 (1 μg/ml in L-15 medium; Sigma-Aldrich, USA) for 5 min, and then washed three times with PBS. The morphology of the nuclei was observed by fluorescence microscopy (Leica, Germany).

### Western Blotting

To obtain the changes of the protein of SGIV MCP, Western blotting was conducted as described previously (Ni et al. 2017). Briefly, cells were lysed using RIPA lysis buffer. The proteins were separated by 10% SDS-PAGE and transferred to polyvinylidene fluoride (PVDF) membranes. The PVDF membranes were blocked with 5% dry milk diluted with TBST for 1 h, and then incubated with anti-β-actin (1:1000 dilution), and anti-SGIV MCP (1:1000 dilution) at 4 °C overnight. The membranes were washed three times with TBST and incubated with goat anti-rabbit IgG and goat anti-mouse IgG antibodies conjugated with horseradish peroxidase (HPR) at room temperature for 1 h. The membranes were washed five times in TBST and incubated with the SuperSignal West Pico chemiluminescent substrate (Thermo, USA), and then exposed to a chemiluminescence imaging analysis system.

### Virus Titer Assay

To determine the effect of miR-122 on SGIV production, the viral titer was evaluated by TCID_50_ analysis. Cells were transfected with miR-122 mimics (100 nM) and infected with SGIV for 12 h. The cells were collected and freeze-thawed three times at − 80 °C. The cell lysates were then serially diluted and used for GS cell infection in 96-well plates. Approximately 6 days after infection, the viral titer was calculated using TCID_50_ analysis.

### AP-1 and NF-κB Activation Analysis

GS cells seeded in 24-well plates were transfected with 100 ng pGL3-luc-NF-κB reporter plasmid, 50 ng SV40 plasmid, and miR-122 mimics/control miRNA (miR-con) (RiboBio, China) which infected with SGIV. Twenty-four hours posttransfection, the cells were collected. The Renilla luciferase and firefly luciferase activities were measured by the Dual-Luciferase Reporter Assay System (Promega, USA).

### Flow Cytometric Analysis of Cell Apoptosis

To explore the role of miR-122 in SGIV-induced apoptosis, miR-122 mimics or miR-con was transfected into FHM cells seeded in 24-well plates with 2 μl of Lipofectamine RNAiMAX (Invitrogen, USA). The cells were collected at 24 h after SGIV infection, and apoptosis was detected by flow cytometry using the Annexin V-FITC Apoptosis Detection Kit (Becton, Dickinson, and Company, USA). The data acquisition and analysis were performed using a flow cytometer system (Beckman Coulter, USA) and FlowJo VX software.

### Caspase-3 Activity Assay

The activity of intracellular caspase-3 was determined by the caspase fluorescent protease assay kit (BioVision, USA). FHM cells transfected with miR-122 mimics or miR-con were for 24 h were infected by SGIV. Twenty-four hours later, cells were collected by trypsinization. The reaction was measured on a multi-label plate reader at 405 nm. The activity of caspase-3 was described as the ratio of the absorbance of the treated sample to nitroaniline to the simulated infected cells.

### Statistical Analysis

All the data are expressed as the mean ± standard error of the mean (SD) and were analyzed with SPSS using one-way analysis of ANOVA followed by Duncan’s test. Significance was set at *P* < 0.05.

## Results

### MiR-122 Is Up-regulated by SGIV Infection

**Fig. 1:**
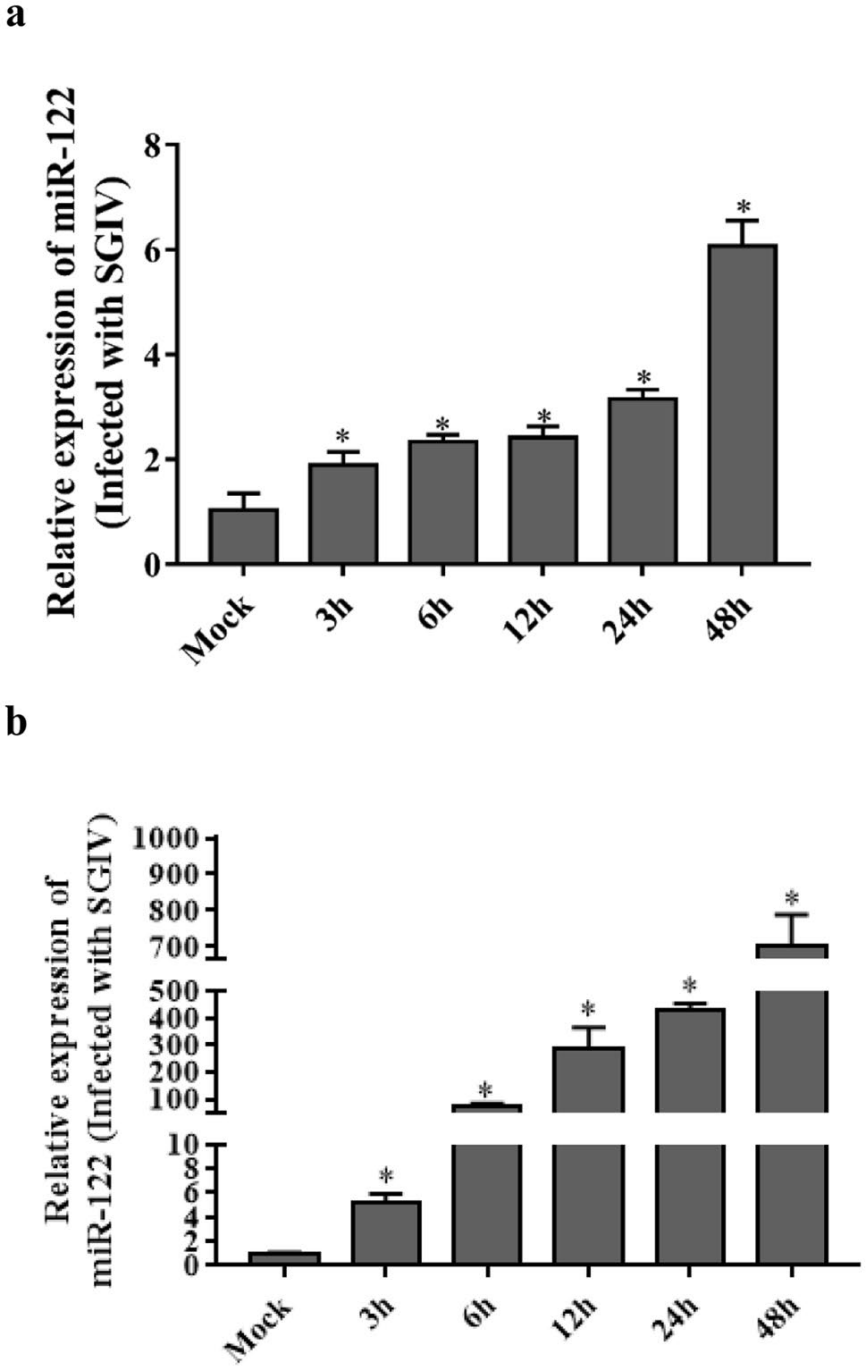
SGIV infection induced miR-122 expression in GS cells. GS cells in six-well plates were infected with an m.o.i. of 0.1 SGIV. Cells were then collected at 3, 6, 12, 24, and 48 h, and RNA purified. Levels of miR-122 were determined by real-time PCR and normalized to U6 **a** or β-actin **b**. Significant differences of miR-122 expression between control and SGIV infection group at each time points is indicated with * (significant increase, *P* < 0.05). All data are expressed as mean ± SE, *N* = 4

*E. coioides* miRNAs were obtained by employing the Solexa deep sequencing approach in our lab (Guo et al. 2015). As shown in Table 2, the sequence of *E. coioides* miR-122 was the same as that from the *D. rerio*, *H. sapiens*, *M. musculus*, *R. norvegicus*, and *B. taurus* except *C. carpio*, indicating that miR-122 is highly conserved. To study the relationship between *E. coioides* miR-122 and SGIV infection, the expression pattern of miR-122 during SGIV infection was examined by qPCR. As shown in Fig. 1, the expression of miR-122 was up-regulated with a peak at 48 h during SGIV infection (*P* < 0.05).

**Table 2:** MiR-122 in different species

miRNA name	Sequence(5′–3′)	Species	miRBase number
eco-miR-122	UGGAGUGUGACAAUGGUGUUUG	*Epinephelus coioides*	Undetermined
dre-miR-122	UGGAGUGUGACAAUGGUGUUUG	*Danio rerio*	MIMAT0001818
ccr-miR-122	UGGAGUGUGACAAUGGUGUUU	*Cyprinus carpio*	MIMAT0026203
hsa-miR-122b-5p	UGGAGUGUGACAAUGGUGUUUG	*Homo sapiens*	MIMAT0000421
mmu-miR-122b-5p	UGGAGUGUGACAAUGGUGUUUG	*Mus musculus*	MIMAT0049852
rno-miR-122-5p	UGGAGUGUGACAAUGGUGUUUG	*Rattus norvegicus*	MIMAT0000827
Bta-miR-122	UGGAGUGUGACAAUGGUGUUUG	*Bos taurus*	MIMAT0003849

### The Effect of miR-122 on SGIV Replication

**Fig. 2:**
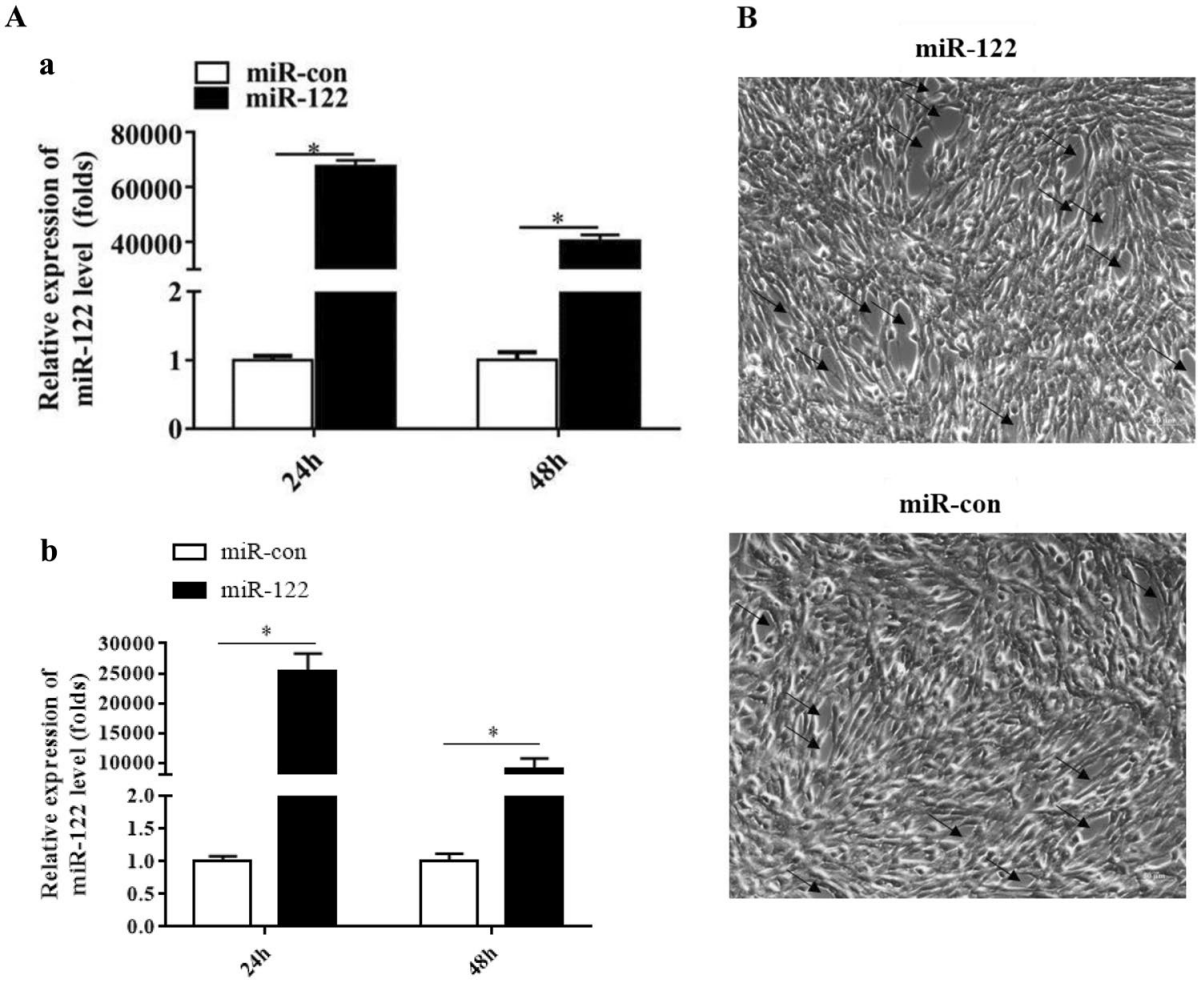
MiR-122 promoted the severity of the CPE induced by SGIV infection in GS cells. **a** The efficiency of miR-122 in the cells after transfection with 100 nM miR-122 mimics (miR-122) or control miR-122 (miR-con). U6 (a) and β-actin (b) were used as an internal reference and the data was shown as multiples. **b** Cell morphology of SGIV-infected miRNA-transfected GS cells. At 12 h after transfection, cells were infected with SGIV at an m.o.i. of 0.1. After 12 h of infection, CPE changes (arrows) were observed under microscope, which was characterized by cell rounding and cell aggregation. Significant differences of miR-122 expression between control and SGIV infection group at each time points is indicated with * (significant increase, *P* < 0.05). All data are expressed as mean ± SD, *N* = 4

To test the efficiency of the miR-122 mimics, the expression of miR-122 at 24 and 48 h was examined after transfection with miR-122 mimics in GS cells. As shown in Fig. 2a, significantly higher expression of miR-122 was detected in cells with the miR-122 mimics for 48 h (*P* < 0.05), showing that the cells can be used to study the effect of over-expression miR-122.

**Fig. 3:**
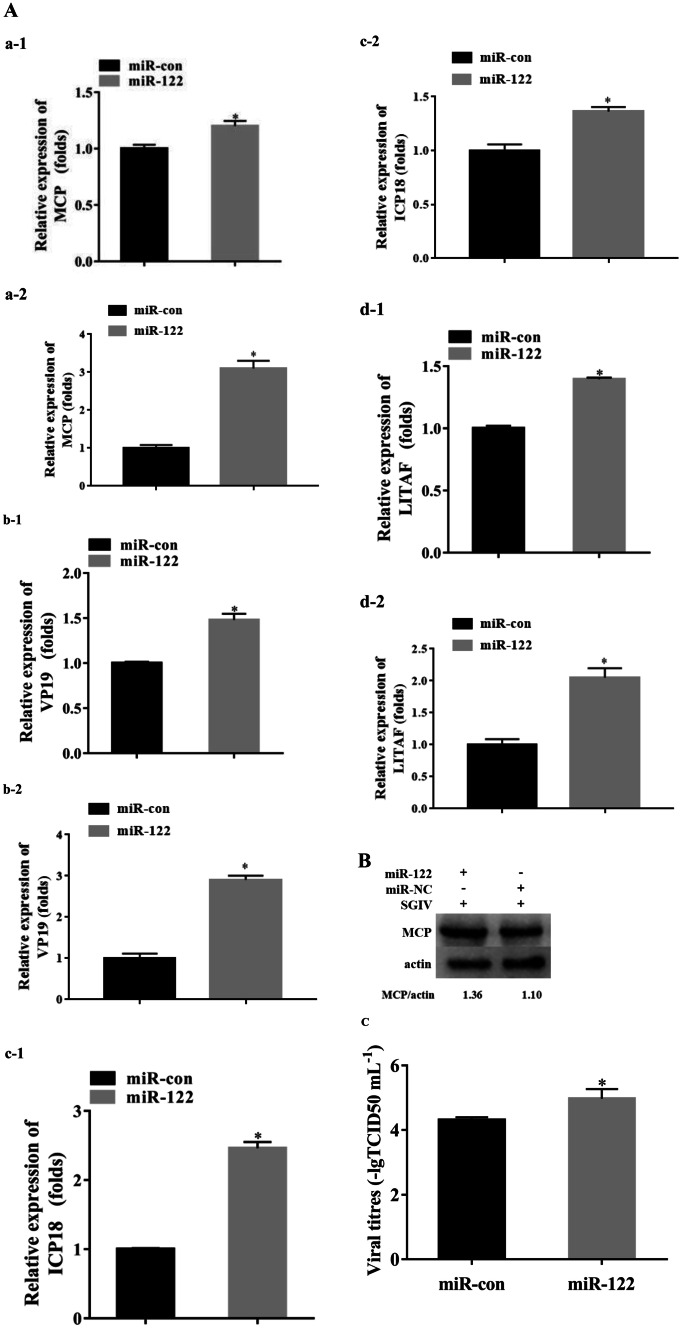
MiR-122 promoted viral replication and the production of infectious progeny SGIV virions. The GS cells transfected with 100 nM miR-122 mimics (miR-122) or control miR-122 (miR-con) for 12 h were infected by an m.o.i. of 0.1 SGIV for 12 h. Subsequently, the viral genes, protein levels and viral titres were detected. **a** The relative expression of four viral genes including MCP, VP19, ICP18, and LITAF were determined by qPCR and normalized to β-actin (a-1, b-1, c-1, and d-1)or 18S rDNA (a-2, b-2, c-2, and d-2). **b** The protein levels of SGIV MCP in the cells transfected with miR-122 or miR-con were determined by western blotting, and β-actin was detected as the internal control. The gray value of SGIV MCP:β-actin was shown. MCP: SGIV, and actin: β-actin. **c** The viral titers in each group were measured using the TCID_50_ method. Significant differences of the genes expression between miR-con and miR-122 group is indicated with * (significant increase, *P* < 0.05).All data are expressed as mean ± SD, *N* = 3

Twelve hours after transfection with the miR-122 mimics, the cells were infected with SGIV, and then, the SGIV-induced cytopathic effects (CPEs) and the viral gene expression were analyzed at 12 h post-SGIV infection. The results showed that the CPE were significantly increased (Fig. 2b). Moreover, the expression of viral genes (MCP, VP19, ICP18, and LITAF) was significantly up-regulated (Fig. 3a); by western blot, the gray value of SGIV MCP:β-actin in the cells transfected with miR-122 mimics was 1.36, and that of the control cells was 1.10 (Fig. 3b), showing that over-expression of miR-122 could enhance the protein synthesis of SGIV MCP. As shown in Fig. 3c, the viral titer, which was used to evaluate viral production, of the miR-122 over-expression cells was significantly higher than that of the control cells (*P* < 0.05).

### The Regulatory Effect Between p38α MAPK and miR-122

**Fig. 4:**
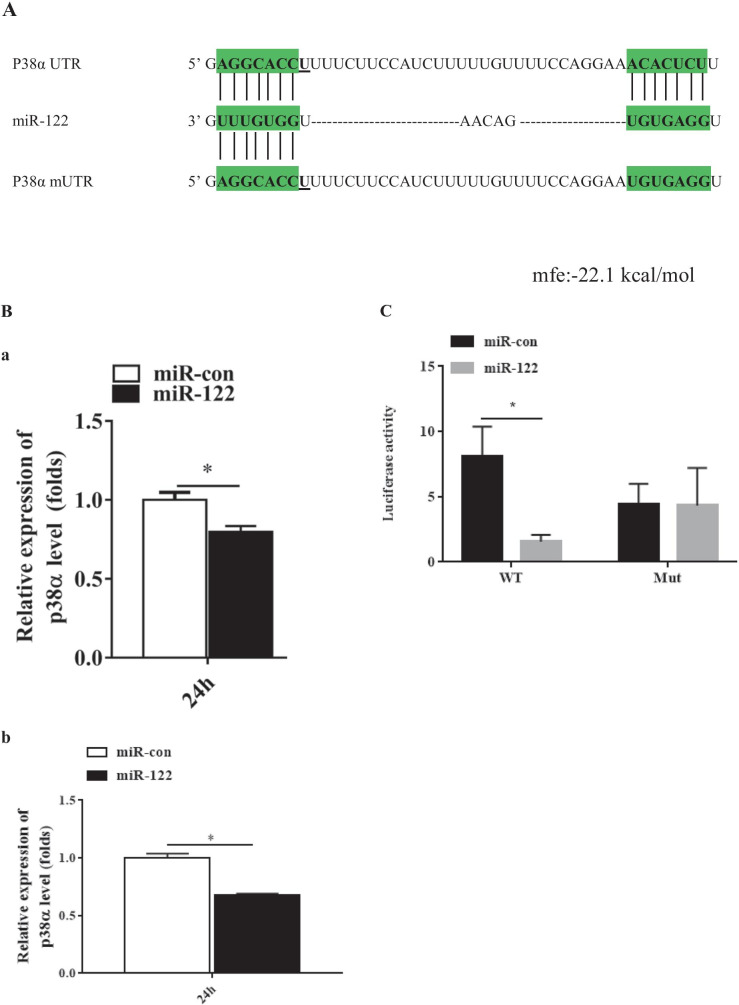
miR-122 targeted the 3′ untranslated region (UTR) of *E. coioides* p38α MAPK. **a** The predicted sites of the grouper target gene p38α MAPK and miR-122 and energy threshold. The binding sites were indicated by green shade. **b** The expression of p38α MAPK in the cells transfected with 100 nM miR-122 mimics (miR-122) or control miR-122 (miR-con), and normalized to β-actin (a)or 18S rDNA (b). **c** The luciferase activity in the cells co-transfected with p38 MAPK (wild or mutant type) and miR-122 mimics. The wild or mutant type UTR of grouper p38α MAPK was cloned into pMIR-REPORT luciferase reporter plasmid, and then co-transfected into GS cells with with 100 nM miR-122 mimics (miR-122). The luciferase activity was determined using reporter gene assay. Significant differences of p38α MAPK expression between miR-con and miR-122 group is indicated with * (significant increase, *P* < 0.05). All data are expressed as mean ± SD, *N* = 3

According to the analysis of bioinformatics tools, p38α MAPK was the target gene, and the binding energy value was − 22.1 kcal/mol. The miR-122 sequence contains a conserved sequence matching the p38α MAPK binding 3′-UTR (Fig. 4a). To study the role miR-122 on p38α MAPK, the p38α MAPK mRNA was examined in the over-expression miR-122 cells by qPCR. As shown in Fig. 3b, the expression of the p38α MAPK was significantly down-regulated in the cells transfected with miR-122 mimics (*P* < 0.05). To verify the relationship of p38α MAPK and miR-122, p38α MAPK 3′-UTR or p38α MAPK mutant 3′-UTR was cloned into a luciferase reporter vector pMIR-REPORT (Ambion, USA). the GS cells transfected with the luciferase reporter containing miR122 mimics and p38αMAPK wild 3′-UTR or p38αMAPK mutant 3′-UTR was analyzed. As shown in Fig. 3c, luciferase activities were significantly reduced in the cells containing p38α MAPK wild 3′-UTR (*P* > 0.05), and there is no significant change in the cells of p38αMAPK mutant 3′-UTR (*P* < 0.05).

### The Effects of miR-122 on Immune Factors

**Fig. 5:**
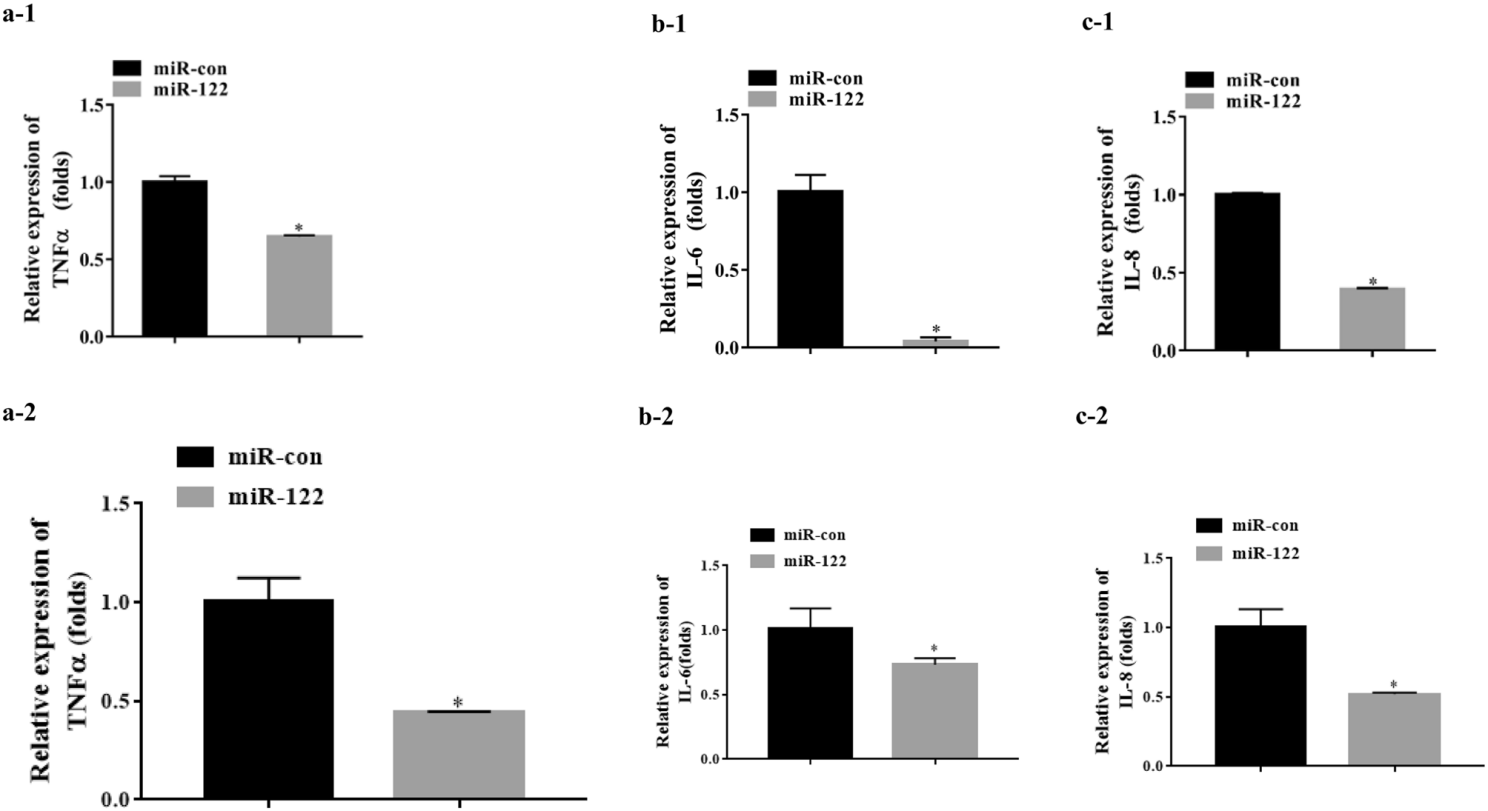
The effect of miR-122 on the transcription of cellular immune factors, TNF-a, IL-6, and IL-8. The GS cells transfected with 100 nM miR-122 mimics (miR-122) or control miR-122 (miR con) for 12 h were infected by SGIV for 12 h. Subsequently, the expression genes were detected and normalized to β-actin (a-1, b-1, c-1) or 18S rDNA (a-2, b-2, c-2). Significant differences of the immune factors expression between miR-con and miR-122 group is indicated with * (significant increase, *P* < 0.05). All data are expressed as mean ± SD, *N* = 4

To verify the roles of miR-122 in immunity, GS cells containing miR-122 mimics or miR-con was infected with SGIV, and the expression of TNF-α, IL-6, and IL-8 was determined. The expression levels of TNF-α, IL-6, and IL-8 were significantly lower than those in the control group (*P* < 0.05) (Fig. 5), suggesting that miR-122 significantly reduced the transcription levels of the immune-related factors.

### MiR-122 Inhibits the Activation of AP-1 and NF-κB

**Fig. 6:**
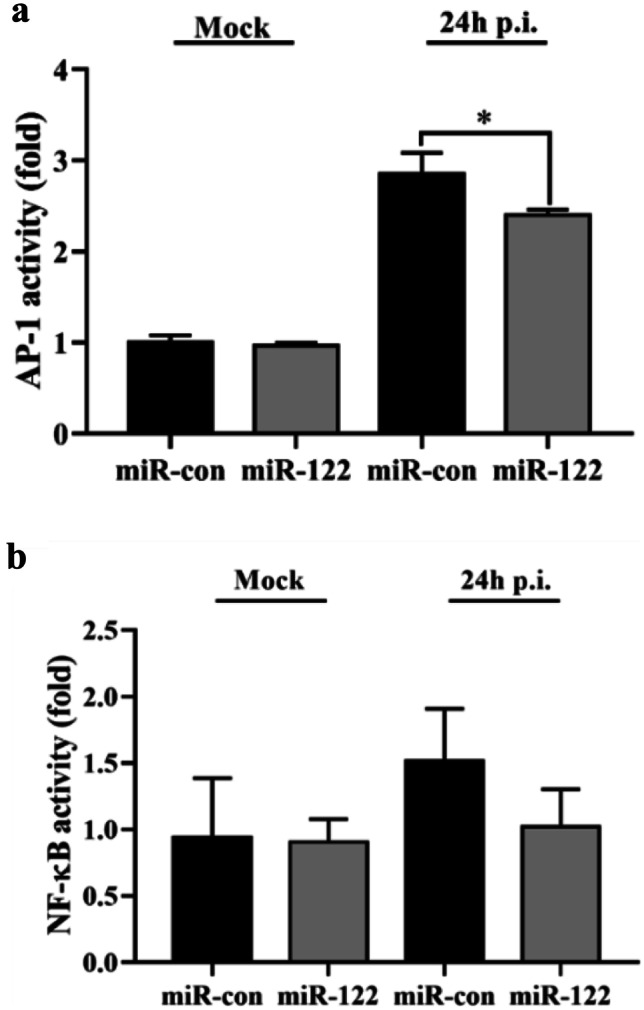
MiR-122 inhibited AP-1 and NF-κB activation. The GS cells transfected with 100 nM miR-122 mimics (miR-122) or control miR-122 (miR con) for 24 h were infected by SGIV for 24 h. Subsequently, the viral genes, protein levels and viral titers were detected. All data are expressed as mean ± SD, *N* = 4, with SPSS using one-way analysis of ANOVA followed by Duncan’s test. *P < 0.05 compared to control group

The effect of *E. coioides* miR-122 on the transcriptional activity of the AP-1 and NF-κB promoters was been investigated. The GS cells transfected with 100 nM miR-122 mimics or control miR-122 at 12 h were infected by SGIV for 12 h. As shown in Fig. 5, the activation of the AP-1 was significantly reduced (*P* < 0.05), and that of NF-κB was just slightly down-regulated in the cells transfected with miR-122 mimics for 24 h compared to the control group (*P* > 0.05) (Fig. 6).

### MiR-122 Inhibits SGIV-Induced Cell Apoptosis

**Fig. 7:**
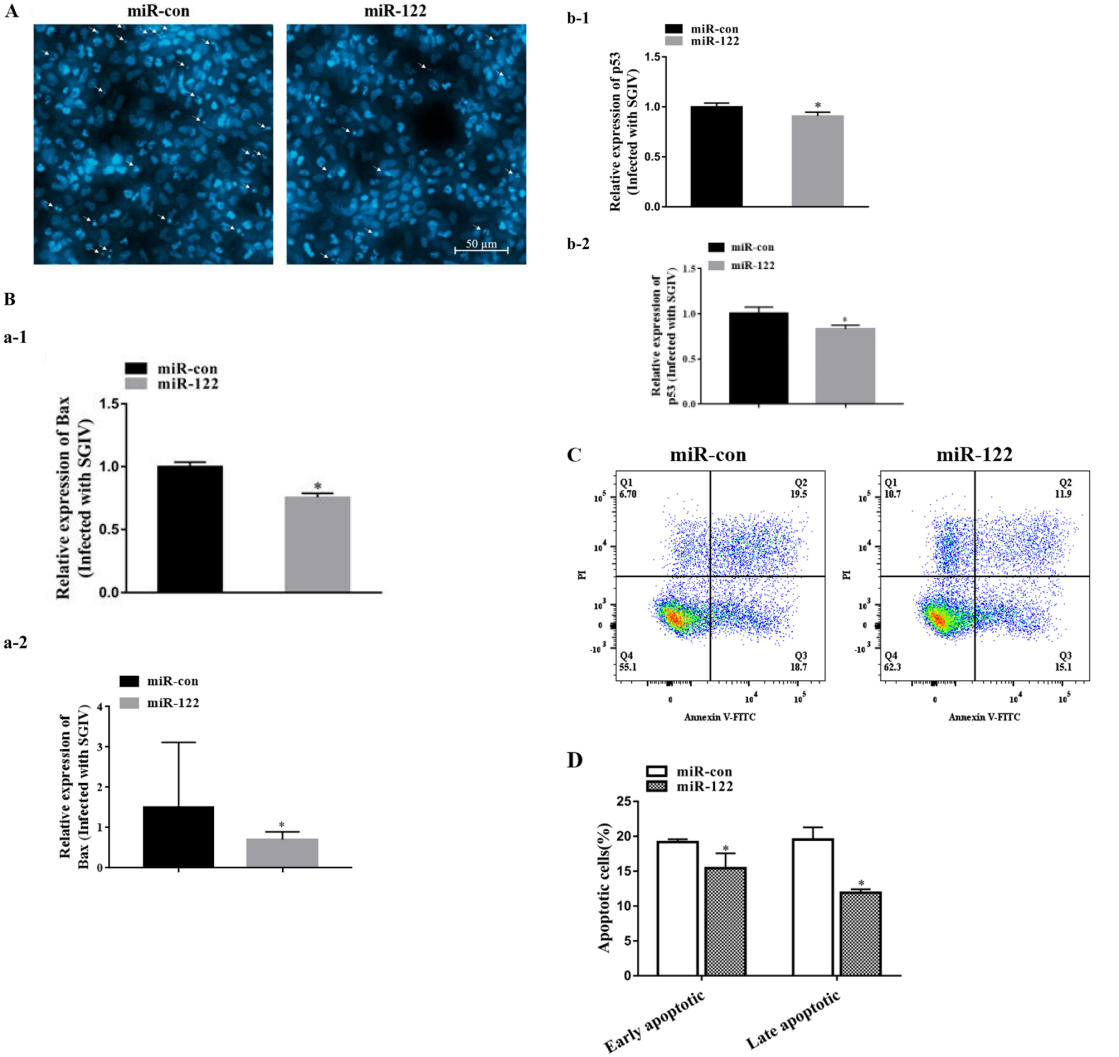
MiR-122 inhibited SGIV-induced the formation of apoptotic bodies and cell apoptosis in FHM cells. The cells transfected with 100 nM miR-122 mimics (miR-122)/control miR-122 (miR-con) for 12 h, and then, the cells were infected with SGIV. Cell apoptosis was analyzed at 12 h after SGIV infection. **a** Visualization of apoptotic bodies in FHM cells. **b** Effect of miR-122 on the transcription of apoptotic factors, Bax and p53. The expressions were normalized to β-actin (Bax: a-1; p53: b-1) or 18S rDNA (Bax: a-2; p53: b-2). **c** Annexin-V+/PI− cells and annexin-V+/PI+ cells are early apoptotic cells and late apoptotic cells, respectively. **d** It is based on the average of three parallel experimental data of graph **c**. Significant differences of the apoptosis between miR-con and miR-122 group is indicated with * (significant increase, *P* < 0.05). All data are expressed as mean ± SD, *N* = 4

SGIV infection can induce apoptosis in FHM cells (Huang et al. 2011a). To investigate the role of miR-122 in SGIV-induced cell apoptosis, the miR-122 mimics/control miR-122 were transfected into FHM cell for 12 h, and then, the cells were infected with SGIV. Cell apoptosis was analyzed at 12 h after SGIV infection. As shown in Fig. 7a, over-expression of miR-122 obviously reduced the SGIV-induced the formation of apoptotic bodies (white arrow); and the expression of the key apoptosis-related factors Bax and p53 was down-regulated (Fig. 7b). The cell apoptosis rate was quantitatively evaluated by performing flow cytometric assays. As shown in Fig. 7c, d, the percentage of early apoptosis in the control cells was 19.17%, and 15.43% occurred in the cells transfected with miR-122 mimics, indicating that miR-122 can significantly inhibit SGIV-induced cell apoptosis.

### The Caspase-3 Activity Detection

**Fig. 8:**
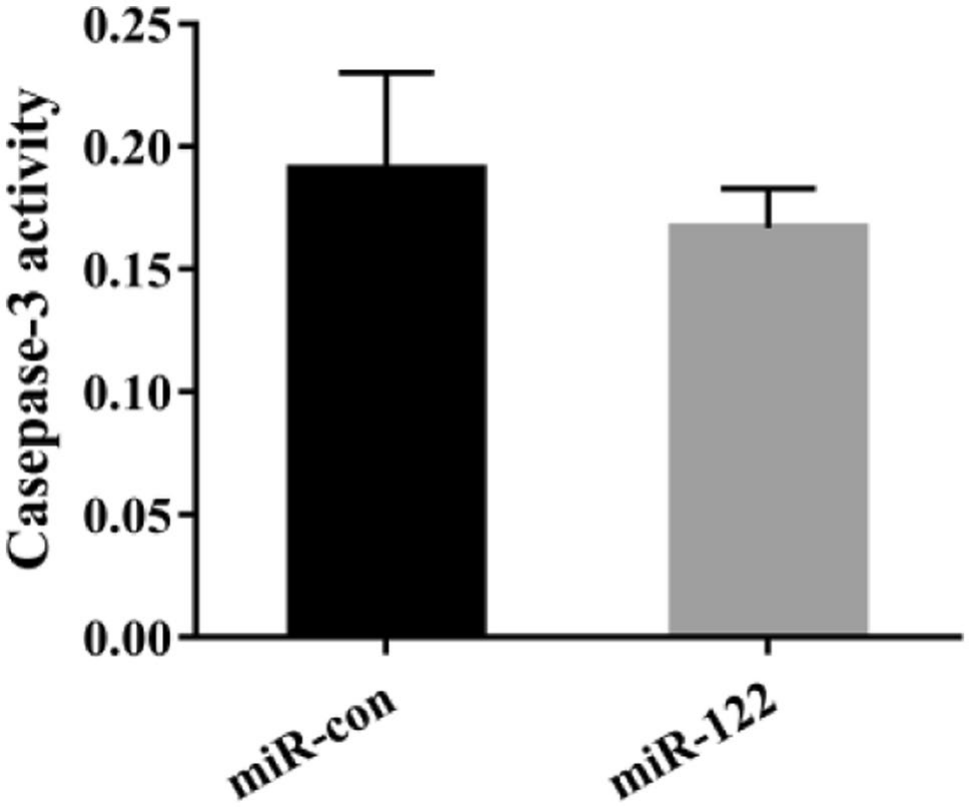
MiR-122 inhibited the caspase-3 after SGIV infection. GS cells transfected with 100 nM miR-122 mimics (miR-122) or control miR-122 (miR-con) were infected by SGIV for 12 h, and the activity of caspase-3 was detected by caspase-3 kit. Significant differences of the casepase-3 activity between miR-con and miR-122 group is indicated with * (significant increase, *P* < 0.05). All data are expressed as mean ± SD, *N* = 4

To explore the effect of miR-122 on SGIV-induced caspase activation, the GS cells transfected with miR-122 mimics or control miR-122 at 12 h were infected by SGIV for 12 h, and the activity of caspase-3 was investigated. As known that SGIV significantly activates caspase-3 activity (Huang et al. 2011b). In this study, the caspase-3 activity in the cells transfected with miR-122 mimics was slightly down-regulated using the caspase fluorescent protease assay kit (*P* > 0.05) (Fig. 8).

## Discussion

MiR-122 in mammals participates in regulating cell growth, apoptosis, innate immunity, and viral translation, infection, and replication (Jangra et al. 2010; Bandiera et al. 2015; Song et al. 2015; Basu et al. 2014; Jopling 2012; Xu et al. 2019; Haldipur et al. 2018). In this study, the roles of *E. coioides* miR-122 in SGIV infection and replication, AP-1 and NF-κB activition, and cell apoptosis were elucidated.

SGIV induced mass mortality in marine fishes, especially in grouper farms. However, the mechanism of SGIV infection needs to be cleared. Many studies have demonstrated the miR-122 in mammals can be involved in the viral infection in higher vertebrates. In this study, miR-122 promoted the replication and proliferation of SGIV, as shown by the significantly enhanced CPE, the up-regulated transcriptional expression levels of virus genes (MCP, VP19, LITAF, and ICP18), and the induced MCP protein levels. In addition, the viral titer was used to evaluate viral production (Ni et al. 2017), and miR-122 significantly increased the TCID_50_, indicating that miR-122 promoted the generation of progeny virus. Similar results have been observed in the mammals: miR-122 promoted HCV virus replication by directly binding to two sites (S1 and S2) in the HCV genomic RNA, and at least partially by stimulating IRES-mediated translation (Jangra et al. 2010); miR-122 induced HEV-1 replication by directly interacting with the target site RdRpc (CACTCC) in the viral genome which provided opportunities for antiviral treatment and management of hepatitis E (Qian et al. 2010).

SGIV induced the typical apoptosis in FHM cells infected by SGIV, as shown by cellular production of DNA ladders and apoptotic bodies, enhanced caspase activity, and increased mitochondrial membrane potential (Song et al. 2004; Huang et al. 2011a, b, c). Apoptosis is a kind of autonomous cell death in order to maintain the stability of the internal environment and is regulated by genes that are essential for the development and function of cells (Green and Reed 1998). In mammals, the apoptotic pathway can be divided into two categories: the mitochondria-mediated intrinsic apoptotic pathway and the death receptor-mediated intrinsic apoptotic pathway (Green and Reed, 1998; Tiwari et al. 2017; Xiong et al. 2014). Mitochondria release cytochrome C, which binds to Apaf-1 and procaspase-9, and then enhances caspase-9 activity, eventually activating downstream factors and causing apoptosis (Tiwari et al. 2017; Reed 2000; Shirjang et al. 2019). The death receptor-mediated internal apoptosis pathway occurs mainly through the TNF receptor family via the transmission of immune signals to cells infected with pathogens, resulting in apoptosis (Elmallah and Micheau 2015; Seol et al. 2015).

In mammals, miRNAs can protect the cells from apoptosis and necrosis by regulating some molecules, endogenous (Bcl-2, Mcl-1), exogenous (TRAIL, Fas, p53), and endoplasmic reticulum stress-induced apoptosis cytokines (Shirjang et al. 2019). miR-122 has been widely reported to participate in the process of apoptosis (Manfe et al. 2012; Guo et al. 2018; Cui et al. 2016). Over-expression miR-122 in bile duct carcinoma cells decreased cell invasion and migration ability, and suppressed cell apoptosis and p53 expression (Wu et al. 2016). MiR-122 mediated by adenoviral vector was induction of apoptosis and cell cycle arrest of cancer cells by inhibiting Bcl-W and CCNG1 expression (Ma et al. 2010). Over-expression of miR-122 significantly reduced N2A cell death, caspase-3 activity, and increased Bcl-2 protein expression in comparison with miR-NC (Guo et al. 2018). The virus damages the host cell and triggers the host cell apoptosis (Ameisen et al. 2013; Mahalingam et al. 2002). And then, apoptosis can reduce the survival space of the virus to limit the viral proliferation and reinfection (Barber and Host 2001; Ludwig et al. 2006). This study revealed that grouper over-expression miR-122 could reduce the number of apoptosis bodies, apoptosis rates, the expression of pro-apoptotic genes (Bax and p53), and slightly inhibit the activity of caspase-3.

In this study, p38α MAPK, one of the important target genes recognized by miR-122, could be mediated by miR-122 during SGIV infection, affects the activity of AP-1 and NF-κB, and the expression of inflammatory signaling molecules, such as TNF-α, IL-6, and IL-8. AP-1 and NF-κB are considered to be involved in immunity and cell apoptosis, and these molecules can also be activated by viral infection (Guo et al. 2016). The production of inflammatory factors is usually caused by NF-κB and AP-1 (Ahn et al. 2016; Yuan et al. 2018). Studies have reported that miR-122 and the immune response play roles in virus replication. MiR-122 is involved in the regulation of various physiology and biological pathways by regulating target genes and antiviral immunity (Jangra et al. 2010; Jopling 2012; Filipowicz and Grosshans 2011; Li et al. 2012). MiR-122 reduces the inhibitory effect on MDA5 by targeting DAK and regulated the RIG-I-like receptor signaling pathway to resist viral infection and enhance the immune response in fish (Han et al. 2018). SVCV viral replication is regulated by inhibiting the MAPK and PI3K inflammation-related signaling pathways (Sun et al. 2020). West Nile virus capsid protein and influenza A virus NS1 protein regulate the PI3K pathway, thereby affecting viral replication (Urbanowski and Hobman 2013; Shin et al. 2007).

In summary, the role of grouper miR-122 during SGIV infection was characterized in this study. The expression level of miR-122 was significantly increased during SGIV infection. Over-expression of miR-122 significantly facilitated SGIV replication in grouper cells. MiR-122 suppressed p38α MAPK-mediated cellular immune response by targeting its 3′ UTR, affect the activities of AP-1 and NF-κB, the expression of inflammatory factors (TNF-α, IL-6, and IL-8) and apoptosis-related genes (Bax and p53), and inhibit the SGIV-induced apoptosis. This study provides new insights into understanding the function of fish miRNAs in virus pathogenesis.
